# Recombinant Fusion Protein Joining E Protein Domain III of Tick-Borne Encephalitis Virus and HSP70 of *Yersinia pseudotuberculosis* as an Antigen for the TI-Complexes

**DOI:** 10.3390/biom8030082

**Published:** 2018-08-25

**Authors:** Vasily Golotin, Nina Sanina, Ludmila Davydova, Natalia Chopenko, Andrey Mazeika, Manuel Roig, Valery Shnyrov, Vladimir N. Uversky, Eduard Kostetsky

**Affiliations:** 1 Department of Biochemistry, Microbiology and Biotechnology, Far Eastern Federal University, Sukhanov St., 8, Vladivostok 690091, Russia; golotin@bk.ru (V.G.); l.vorobushek@mail.ru (L.D.); natali_1389@mail.ru (N.C.); nikandrei@inbox.ru (A.M.); kostetskiy.yeya@dvfu.ru (E.K.); 2 Laboratory of Marine Natural Compounds Chemistry, G.B. Elyakov Pacific Institute of Bioorganic Chemistry, FEB RAS, Prospect 100 let Vladivostoku, 159, Vladivostok 690022, Russia; 3 Departamento de Química Física, Facultad de Ciencias Químicas, Universidad de Salamanca, Plaza de los Caìdos s/n, 37008 Salamanca, Spain; mgr@usal.es; 4 Departamento de Bioquímica y Biología Molecular, Facultad de Biología, Universidad de Salamanca, Plaza Doctores de la Reina s/n, 37007 Salamanca, Spain; shnyrov@usal.es; 5 Department of Molecular Medicine and USF Health Byrd Alzheimer’s Research Institute, Morsani College of Medicine, University of South Florida, 12901 Bruce B. Downs Blvd. MDC07, Tampa, FL 33612, USA; vuversky@health.usf.edu; 6 Laboratory of New methods in Biology, Institute for Biological Instrumentation, Russian Academy of Sciences, Pushchino 142290, Moscow Region, Russia

**Keywords:** domain III, envelope protein, HSP70, fusion protein, TBEV, flavivirus vaccine, differential scanning calorimetry, intrinsic fluorescence

## Abstract

Domain III (DIII) of the tick-borne encephalitis virus (TBEV) protein E contains epitopes, which induce antibodies capable of neutralizing the virus. To enhance the immunogenicity of this protein, which has a low molecular weight, the aim of the present work was to express, isolate, and characterize a chimeric protein based on the fusion of the bacterial chaperone HSP70 of *Yersinia pseudotuberculosis* and EIII (DIII + stem) as a prospective antigen for an adjuvanted delivery system, the tubular immunostimulating complex (TI-complex). The chimeric construction was obtained using pET-40b(+) vector by ligating the respective genes. The resulting plasmid was transformed into DE3 cells for the heterologous expression of the chimeric protein, which was purified by immobilized metal affinity chromatography (IMAC). ELISA, differential scanning calorimetry, intrinsic fluorescence, and computational analysis were applied for the characterization of the immunogenicity and conformation of the chimeric protein. Mice immunization showed that the chimeric protein induced twice the number of anti-EIII antibodies in comparison with EIII alone. In turn, the incorporation of the HSP70/EIII chimeric protein in the TI-complex resulted in a twofold increase in its immunogenicity. The formation of this vaccine construction was accompanied by significant conformational changes in the chimeric protein. Using HSP70 in the content of the chimeric protein represents an efficient means for presenting the main antigenic domain of the TBEV envelope protein to the immune system, whereas the incorporation of this chimeric protein into the TI-complex further contributes to the development of a stronger immune response against the TBEV infection.

## 1. Introduction

Tick-borne encephalitis (TBE) is a viral infection that affects the main parts of the brain and spinal cord of the central nervous system, as well as spinal nerve roots and peripheral nerves. As a result, a TBE infection may result in long-lasting neurological complications, or even death [1]. There is no specific antiviral medication against TBE infection, and the only currently available treatment for it is restricted to symptomatic therapy. However, TBE can be prevented successfully by vaccination. Currently available commercial vaccines based on the inactivated whole virus show mild and transitory side-effects [2]. Although this type of vaccine is successful, the manufacturing of inactivated TBE vaccines is associated with processing a large number of dangerous pathogens. Therefore, the development of new vaccines with a safe production process that could cause prolonged immunity is much-needed [3,4]. Highly effective and safe vaccines based on the recombinant subunits of viral proteins might represent a promising alternative to the existing vaccines based on the inactivated virus.

Tick-borne encephalitis virus (TBEV) is a spherical virus of the *Flavivirus* genus. Virion particles are covered with a glycoprotein coat representing a continuous protein lattice of the homodimers of an envelope protein (protein E) [5]. The protein E is a 496 residue-long class II fusion protein that plays a key role in the processes of virus particle assembly, virion budding in the endoplasmic reticulum of the host cells, binding of the virus to the cell surface, and fusion of the viral and the host cell membranes. Hence, this protein determines the tropism of the virus. Each monomer of protein E consists of three domains (domains I, II, and III), stem, and a hydrophobic anchor that holds the protein in the lipid membrane of the virion. According to the flavivirus convention, the stem and the hydrophobic anchor form the C-terminal domain IV of the protein E [6]. Domain III (DIII) is one of three N-terminal domains that form an ectodomain containing about 400 residues, and is located outside the viral membrane. DIII includes the most important epitopes of protein E, which induces antibodies neutralizing the virus and prevents the pH-induced conformational changes of E-proteins required for receptor binding [7]. However, DIII is not immunogenic due to its low molecular weight (MW 16 kDa). One of the strategies for increasing the immunogenicity of this protein is the creation of chimeric (hybrid) recombinant proteins with specified properties and with decreased DIII toxicity for bacterial host cells [8]. Heat shock proteins (HSPs) serve as promising fusion partners due to their remarkable effects on the immune system [9]. However, even large proteins are often weak antigens that need adjuvants. Our previous studies have shown that tubular immunostimulating complexes (TI-complexes) enhance the immune response against different antigens, such as porin OmpF of the enterobacteria *Yersinia pseudotuberculosis* [10], the HA1 subunit of the Influenza A H1N1 hemagglutin (A/California/04/2009(H1N1)) [11], and the recombinant hemagglutinin monomer of the influenza A virus H1/N1 [12].

The nanoparticulate TI-complex is an adjuvanted antigen delivery system consisting of cholesterol, triterpene glycoside cucumarioside A_2_-2 (CDA), and glycolipid monogalactosyldiacylglycerol (MGDG) isolated from marine macrophytes. MGDG forms a lipid matrix for the protein antigen incorporated into TI-complexes. Its fatty acid composition and microviscosity, which depend on the taxonomic position of marine macrophytes, can differently influence the conformation and immunogenicity of a protein antigen [10].

The aim of the present work was to express, isolate, and characterize a chimeric HSP70/EIII protein based on the fusion of the bacterial HSP70 of *Y. pseudotuberculosis* and EIII (DIII + stem) domains of the TBEV E protein as a prospective antigen for the TI-complexes and the development of an anti-TBE subunit vaccine.

## 2. Materials and Methods

### 2.1. Construction of Chimeric Plasmids

The plasmids for the expression of the recombinant proteins EIII, HSP70, and chimeric HSP70/EIII protein were constructed using the pET-40b(+) vector (Novagen, Gibbstown, NJ, USA). For the amplification of the Y. pseudotuberculosis HSP70 gene, the chromosomal DNA of the *Y. pseudotuberculosis* strain 488, Encyclo Taq polymerase (Evrogen, Moscow, Russia), the gene-specific upstream primer dnaK-X-Nco-dir: 5′-ATATCCA TGGCGATGGGTAAAATTATTGGTATCGAC-3′, and the downstream primer dnaK-X-Sac-rev: 5′-TATAGAGCTCGCTTTTTTGTCTTTTACTTCTTCGAATTC-3′ were used. The recombinant plasmid 40HSP70 was obtained by ligation of the HSP70 gene into the pET-40b(+) at the restriction sites of NcoI and SacI. The resultant 40HSP70 plasmid was used for the expression of the HSP70 protein and was also used for the subsequent cloning of the EIII protein.

For the EIII gene amplification, cDNA of TBEV strain Dalnegorsk, Encyclo Taq polymerase, the upstream primer including the flexible linker (G_4_S)_3_ E3-X-Sac-L-dir: 5′-TATAGAGCTCGGGTGGT GGTGGTTCTGGTGGTGGTGGTTCTGGTGGTGGTGGTTCTGGTCTTACATACACAATGTGCGACAAGACGAAATTCAC-3′, and the downstream primer, E3-X-Sal-stop-rev: 5′-TATAGTCGACTT AGTTAAAGGCACCGCCAAGAACTGT-3′ were used. The TBEV cDNA was kindly provided by the Institute of Epidemology and Microbiology, Vladivostok, Russia [13].

The plasmids 40HSP70/EIII and 40/EIII were obtained by ligation of the EIII gene into 40HSP70 and pET-40b(+), respectively, at the restriction sites of SacI and SalI. The resultant 40HSP70/EIII and 40/EIII plasmids were used for the expression of chimeric HSP70/EIII protein and the recombinant domain III of TBEV protein E. Since the EIII protein was not expressed separately due to its high toxicity for *Escherichia coli* cells, we used the EIII protein obtained using the cell-free continuous exchange system, as described previously [8]. All used primers are shown in Table 1.

### 2.2. Expression of the Recombinant Plasmids

For the expression of all plasmids used in this study, the *E. coli* Rosetta (DE3) cells were applied. *E. coli* cells transformed with the recombinant plasmids were grown on a Luria-Bertani (LB) agar (Helicon, Moscow, Russia) plate containing 100 µg/mL kanamycin overnight at 37 °C. A single colony was picked and grown with agitation at 200 rpm in 20 mL of LB medium containing 100 µg/mL of kanamycin at 37 °C overnight, then transferred into 0.5 L of MX medium (Medium for eXpression) [14]. When the cell density reached an OD_600_ of 0.8, 0.2 mM of inducer IPTG was added to the bacterial culture, and the incubation was continued at 18 °C up to 24 h with agitation at 250 rpm. *E. coli* Rosetta (DE3) cells were transformed with pET-40b(+) (Novagen, Gibbstown, NJ, USA) as a control of the expression and for the purification of the plasmid.

### 2.3. Recombinant Protein Purification

The purification of all recombinant proteins used in this study was carried out at 8 °C. Harvested *E. coli* cells were resuspended in 300 mL of 10 mM sodium phosphate buffer (SPB), pH 7.7 and 0.1 mg/mL DNAse and totally lysed by sonication, then centrifuged at 10,000× *g* for 10 min at 4 °C. The supernatant was incubated at 37 °C for 1 h, then cooled on ice. A total of 10 mM imidazole was added, and the resulting extract was centrifuged again at 10,000× *g* for 30 min at 4 °C. After centrifugation, supernatant was applied to a Hi-Scale 26/40 (GE Healthcare Europe GmbH, Barcelona, Spain) column containing Ni-charged IMAC Sepharose HP (GE). The column was washed by a buffer containing 10 mM SPB, pH 6.8, 1 M NaCl, and 25 mM imidazole. Then, the column was washed again by the buffer containing 10 mM SPB, pH 5.5, and 25 mM imidazole. Elution was performed by linear gradient of imidazole concentration up to 250 mM in the same buffer. Fractions containing the recombinant protein were pooled and dialyzed overnight against 10 mM SPB, pH 7.3, with 150 mM NaCl. All steps were followed by SDS-PAGE in 12.5% gel [15] to control the efficiency of expression and purification. The total protein concentration of the collected fractions was determined by the Bradford protein assay [16] using bovine serum albumin (BSA) as a standard. All biochemical reagents were purchased from Helicon, Thermo Scientific (Waltham, MA, USA), and Sigma-Aldrich (Saint Louis, MO, USA).

### 2.4. Fluorescence Spectroscopy

Steady-state fluorescence measurements of chimeric HSP70/EIII protein were performed on a RF6000 spectrofluorimeter (Shimadzu, Kyoto, Japan). Fluorescence was excited at 296 nm with excitation and emission slit widths of 3 nm. Fluorescence was measured in the range of 300–400 nm. HSP70/EIII fluorescence measurements were carried out for protein solutions with an optical density of less than 0.2 at 280 nm in order to avoid the inner filter effect. Emission spectra were corrected for baseline and instrumental spectral sensitivity.

### 2.5. Calorimetry

Calorimetric studies were conducted using a SCAL-1 differential scanning microcalorimeter (Scal Co. Ltd., Pushchino, Russia) as described previously [10]. The reversibility of the thermal transitions was verified by checking the reproducibility of the calorimetric profiles at the second heating of the sample immediately after cooling it from the first scan. The temperature dependence of the molar heat capacity of the samples was further analyzed and plotted using a Windows-based software package (ORIGIN) supplied by MicroCal (Northampton, MA, USA).

### 2.6. Preparation of TI-Complexes

TI-complex was prepared as described [17]. A total of 5 mg of MGDG isolated from marine macrophyte *Ulva lactuca* as described in [18] was dissolved in 1 mL of chloroform; 5 mg of cholesterol was dissolved in 1 mL of chloroform; and 4 mg of CDA isolated from marine invertebrate Cucumaria japonica according to [17] was dissolved in 1 mL of distilled water. Then, 132 μL of the MGDG solution and 66 μL of the cholesterol solution were evaporated to dryness under a stream of air at a temperature of 60 °C. A total of 84 μL of a CDA solution was added to this dry residue. A total of 416 μL of PBS of pH 7.2 was further added to this mixture to adjust the MGDG and cholesterol concentrations to 2 mg/mL. The resulting suspension was sonicated for 5 min on a SONOPULS HD 2070 (Bandelin, Germany) ultrasonic disintegrator at 10% maximum power, using the mode of 0.7 s—work; 0.3 s—interval. After sonication, the preparations were stored at 4 °C for no longer than 24 h.

To obtain the TI-complex containing 10 μg of recombinant protein per 10 μg of CDA, 165 μL of the TI-complex preparation was collected immediately after sonication and combined with a 220 μL of the recombinant protein solution in PBS, pH 7.2 at a concentration of 0.5 mg/mL and 715 μL of PBS, pH 7.2. This mixture was vortexed for 1 min. The preparations were stored at 4 °C for no longer than 24 h.

### 2.7. Animals and Immunization

Adult CD1 mice (males) with a weight of 18–21 g were obtained from the Branch of Shemiakin and Ovchinnikov Institute of Bioorganic Chemistry, Russian Academy of Science Animal Breeding Facility, Russia. The animals were maintained in a vivarium of the Pacific Institute of Bioorganic Chemistry, Far East Branch of the Russian Academy of Sciences (FEB RAS), under standard conditions with unlimited access to food and water. All experiments with animals were conducted in accordance with the provisions of Directive N 2010/63/EC of the European Parliament and of the Council of the European Union “On protection of animals used for scientific purposes”. This investigation was approved by the Local Ethics Committee of Pacific Institute of Bioorganic Chemistry, FEB RAS, with the project identification code 0117; the date of approval was 16 January 2017. Four groups, with ten mice in each group, were immunized subcutaneously twice at an interval of 14 days with the same regimen for all antigens. Mice in the first to the third groups were injected with 20 μg of the EIII protein, 20 μg of the HSP70-EIII protein, 20 μg of the HSP70-EIII protein incorporated in TI-complex. Mice in the control group were injected with 1X PBS. The experiment was terminated 28 days after the first immunization.

### 2.8. ELISA and Statistical Analyses

The content of antibodies against the EIII protein was estimated in the mice blood serum using ELISA, applying anti-mouse IgG labeled with the horseradish peroxidase (N.F. Gamaleya Research Institute of Epidemiology and Microbiology) according to [19]. The sera of the animals injected with PBS served as controls. The sensitization of the solid surfaces was carried out by introduction of the recombinant EIII protein solution into the wells of 96-well microtiter plates according to [17]. The optical density of the antibody samples was estimated using an Elx808IU microplate photometer (BioTek Instruments, Winuski, VT, USA) and expressed as absorption at a wavelength of 450 nm (chromogen-3,3,5,5′-tetramethylbenzidine). The dilution of the serum was 1:800. The results were expressed as means ± S.D. of ten observations. The differences among the means were analyzed by parametric analysis, using the Student’s *t*-test. A value *p* < 0.05 was considered as statistically significant.

### 2.9. Computational Analysis of the Intrinsic Disorder Predisposition of the Chimeric HSP70/EIII Protein

The peculiarities of the intrinsic disorder distribution within the amino acid sequence of the chimeric HSP70/EIII protein were analyzed by a set of commonly used per-residue disorder predictors, such as PONDR FIT [20], PONDR VLXT [21], PONDR VSL2 [22], PONDR VL3 [23], and two forms of IUPred suitable for prediction of short and long intrinsically disordered regions, IUPred_short and IUPred_long, respectively [24]. These predictors were selected based on their specific sensitivities to different features associated with intrinsic disorder. PONDR VSL2B is one of the more accurate stand-alone disorder predictors [25,26,27], PONDR VL3 is characterized by high accuracy in predicting long intrinsically disordered regions [23], PONDR VLXT is known to have high sensitivity to local sequence peculiarities and can be used to identify disorder-based interaction sites [28], whereas the metapredictor PONDR-FIT is moderately more accurate than each of the component predictors [20], PONDR VLXT [22,28], PONDR VSL2 [22], PONDR VL3 [23], FoldIndex [29], IUPred [24], and TopIDP [30]. IUPred was designed to recognize intrinsically disordered protein regions (IDPRs) from the amino acid sequence alone based on the estimated pairwise energy content [24,31]. We also analyzed the mean disorder propensity for these proteins by averaging the disorder profiles of individual predictors. The use of consensus for the evaluation of intrinsic disorder is based on empirical observations showing that such an approach usually increases the predictive performance compared to the use of a single predictor [20,27,32,33]. In these analyses, predicted disorder scores above 0.5 were considered to correspond to disordered residues and regions.

## 3. Results and Discussion

For all constructs analyzed in this study, the commercial vector pET-40b(+) was used. This plasmid has important features, such as the presence of the gene encoding redox protein DsbC, which promotes the correct protein folding of the recombinant protein. It also has an N-terminal signal sequence, which allows the direction of the recombinant protein to the cell periplasm.

**Figure 1:**
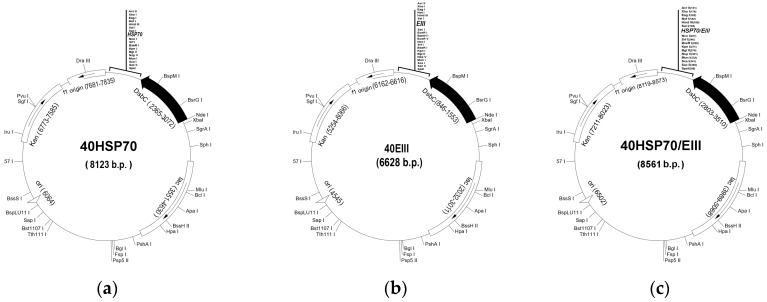
The recombinant plasmid maps encoding recombinant HSP70 (**a**), EIII (**b**) and hybrid HSP70/EIII (**c**).

To construct three recombinant plasmids using the commercial vector pET-40b(+), two PCR products were created, the amplified 450 base pair (b.p) fragment of the gene encoding domain III of the TBEV E protein and gene *dnaK* of 2100 b.p. corresponding to the mature form of HSP70 of *Y. pseudotuberculosis.* These two PCR products were inserted between the SacI/SalI and NcoI/SacI-restriction fragments of the pET-40b(+) vector, respectively (Figure 1).

To improve the efficiency of ligation, the restricted pET-40b(+) vectors were treated by alkaline phosphatase CmAP for dephosphorylation of plasmid ends to prevent the self-ligation of the vector [34]. The resultant plasmids 40EIII and 40HSP70 were obtained. To construct chimeric plasmid 40HSP70/EIII encoding a hybrid protein containing mature HSP70 and protein EIII, the plasmid 40HSP70 was digested and the gene encoding for EIII was inserted between the SacI/SalI-restriction fragments of the plasmid. Previously developed expression conditions and the medium MX were used for the expression of all plasmids obtained [14]. Using the method described in [14], the soluble forms of HSP70 and HSP/EIII were obtained (Figure 1).

An empty plasmid pET-40b(+) was also expressed and a part of the DsbC protein was obtained as a control of the expression and was also used for further purification. The control protein DsbC is a part of all proteins obtained in our study, and is required as a control for other experiments. This protein is a chaperone and a disulfide isomerase. It promotes the correct folding of recombinant proteins in the process of post-translational modification during heterologous expression and also has an *E. coli* signal sequence in the N-terminal part that directs the synthesized protein to the periplasmic space of the host cell. After expression of the 40EIII plasmid, the EIII protein overexpression band was not detected in all protein fractions. This observation indicated that the EIII protein, which is toxic to the *E. coli* host cells, obviously cannot be expressed separately from other proteins, which probably masks its toxic effects on producing cells. Therefore, we used the EIII protein produced using the continuous exchange cell-free system, as described earlier [8].

**Figure 2:**
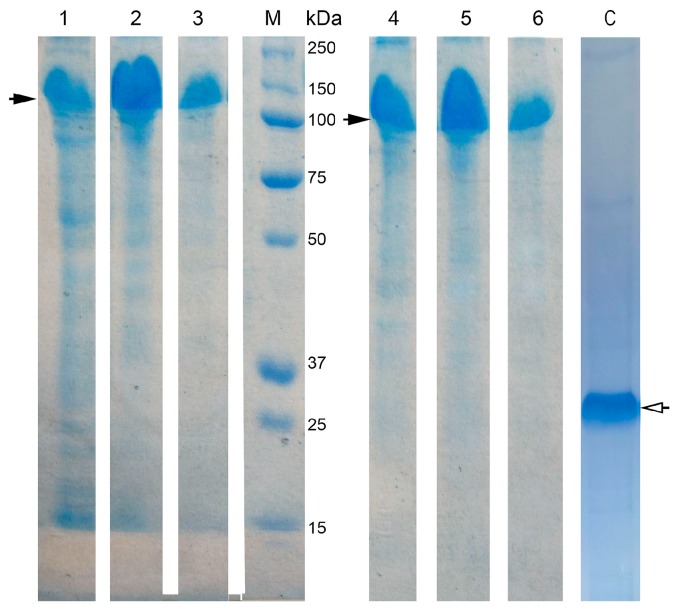
SDS-PAGE of the soluble recombinant protein fractions after expression and purification. 1—crude soluble cell extract fraction after the expression of 40HSP70/EIII plasmid; 2 and 3—purified hybrid HSP70/EIII 40 µg and 20 µg *per* lane, respectively; M—molecular weight marker of proteins (Bio-Rad); 4—crude soluble cell extract fraction after expression of 40HSP70 plasmid; 5 and 6—purified recombinant HSP70 (40 µg and 20 µg *per* lane, respectively); C—purified control protein DsbC. Empty arrow—control band (DsbC-overhang, lane C); filled arrows—bands of the recombinant HSP70/EIII and HSP70 (lanes 1–6).

The conditions for the efficient one-step purification of the expressed proteins were developed. All recombinant proteins analyzed in this study have an N-terminal 6×His-tag that permitted using the metal-affinity chromatography as the most efficient one-step purification. After purification, proteins of 95% purity were obtained according to SDS-PAGE (Figure 2). The total yields of the purified proteins were 203 mg for HSP70 and 158 mg for HSP70/EIII from 1 L of bacterial culture. The DsbC protein was also purified in small quantities to control its presence/absence in the fractions of HSP70 and HSP70/EIII proteins. According to the SDS-PAGE analysis, the apparent molecular mass values of the purified proteins were 99 kDa for HSP70 (70 kDa HSP70 with the 29 kDa DsbC appendage) and 116 kDa for HSP70/EIII (87 kDa HSP70/EIII with the 29 kDa DsbC appendage).

**Figure 3:**
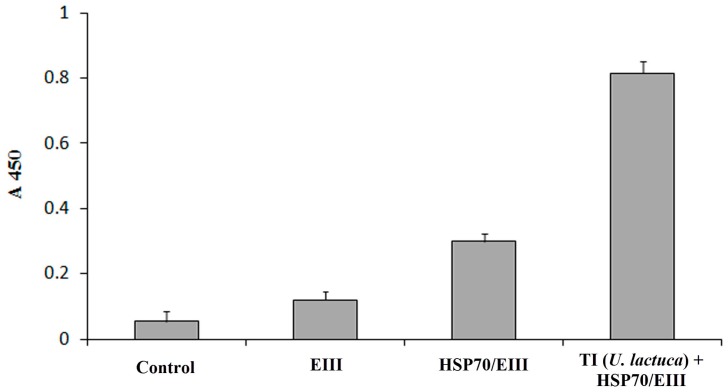
The levels of anti-EIII antibodies in blood sera of mice injected with EIII, chimeric HSP70/EIII protein and HSP70/EIII incorporated in tubular immunostimulating complex (TI) based on monogalactosyldiacylglycerol (MGDG) from *Ulva lactuca* (TI (*U. lactuca*) + HSP70/EIII). Control—mice injected with PBS. Bars represent mean ± S.D. values. Y axis represents absorbance at 450 nm (A450). TI-complex comprised CDA, cholesterol and MGDG at weight ratio of 2:2:4.

The mice immunization experiment showed that the individual HSP70/EIII protein induced a two times higher production of anti-EIII antibodies in comparison with the low-molecular EIII protein (16 kDa) alone (Figure 3). In turn, the incorporation of the chimeric protein in the TI-complex resulted in two-fold increase in the levels of the anti-EIII antibodies.

The use of the *Y. pseudotuberculosis* HSP70 as an EIII companion protein likely leads to better antigen presentation [35] and increases the EIII immunogenicity. This is similar to the HSP70 from *Mycobacterium tuberculosis*, which proved its effectiveness as an adjuvant enhancing the immunogenicity of weak antigens of various natures. Proteins fused to HSP70 elicit strong humoral and cellular immune responses [36,37]. In addition, HSP70 is able to insert into a lipid membrane [38] and thereby serves as an anchor of the chimeric HSP70/EIII protein. The binding of HSP70 to lipid membranes depends on the lipid composition [39] and provides a means for the indirect regulation of the EIII conformation and optimized presentation of DIII in the HSP70/EIII content.

**Figure 4:**
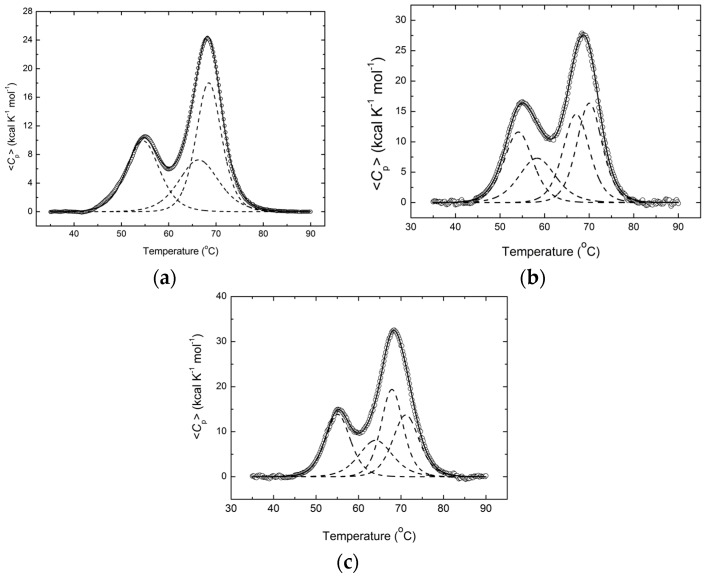
Temperature dependence of the excess molar heat capacity of Hsp70 (**a**), Hsp70/EIII (**b**) and Hsp70/EIII + MGDG from *U. lactuca* (**c**) in sodium phosphate buffer, pH 7.2*.* The symbols are experimental data, while the dashed lines resulted from fitting the data to the two-state model, and solid lines are their sum. The scan rate was 1 K/min. Protein concentration was 0.5 mg/mL.

Differential scanning calorimetry (DSC) was used to investigate the integral changes in the biological macromolecules associated with the release or absorption of heat. The extent of the reversibility measured by the relative area recovery seen on the second scan of HSP70 and HSP70/EIII depended on the temperature at which the first scan was terminated before cooling the samples in preparation for the second scan. In all cases, the low-temperature transitions (Figure 4) were fully reversible. If heating was terminated at temperatures before the second peak maximum, then the second scan showed about 80–90% reversibility, but melting became irreversible when the heating was conducted to higher temperatures. We found, however, that the HSP70 and HSP70/EIII melting results obtained in this work were practically independent of the scan rate, suggesting that the denaturation process was not kinetically determined. Therefore, we concluded that the data could be analyzed semi-quantitatively using thermodynamic models [40,41].

The apparent molar heat capacity of the HSP70, HSP70-EIII, and HSP70-EIII + MGDG samples and the results of the deconvolution conducted by the software provided by MicroCal under the assumption of a two-state model of unfolding are provided in Figure 4, whereas Table 2 summarizes the thermodynamic data calculated for the individual transitions.

Figure 4 and Table 2 show that thermal denaturation of HSP70 represents a complex process, based on the complex shape of the calorimetric curve containing two clearly visible peaks at 53.8 and 68.2 °C, whereas the deconvolution of this calorimetric curve revealed the presence of three peaks positioned at 54.6, 66.4, and 68.5 °C. It is interesting that the calorimetric curve of a structurally and functionally close HSP90 isolated from porcine brain consisted of two peaks at 53.8 and 63.2 °C, and the presence of only two transitions was further supported by the deconvolution of that calorimetric curve [42]. Consequently, the third peak at 68.5 °C (*T*_m_4), found as a result of the deconvolution of the calorimetric curve corresponding to the recombinant HSP70, seems to arise as a result of the DsbC domain melting, which was absent in the HSP90 from the porcine brain.

Although the thermogram of HSP70/EIII contained two heat absorption peaks, the deconvolution of the resulting calorimetric curve into elementary components revealed the presence of four independently melting regions (calorimetric domains), instead of three such domains found in HSP70. Three peaks at 54.2, 67.1, and 70.1 °C corresponded to the melting of HSP70 within the HSP70/EIII chimera. It can be seen that the addition of EIII resulted in some minor stabilization of the chimeric protein, which, in its isolated form, had heat absorption peaks at 54.6, 66.4, and 68.5 °C, respectively. Hence, an additional peak at 58.4 °C (*T*_m_2) likely corresponded to the melting of EIII in the content of HSP70/EIII, since this peak was absent in the melting of the isolated HSP70 construct.

**Figure 5:**
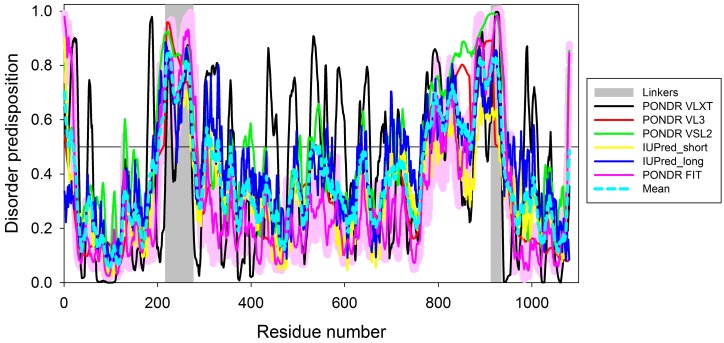
Evaluation of the intrinsic disorder propensity of the HSP70/EIII chimeric protein by a set of disorder predictors. Bold dashed dark cyan line shows the mean disorder propensity calculated by averaging the disorder profiles of individual predictors. Light pink shadow around the PONDR FIT curve shows the error distribution. Linkers connecting the DsbC domain, HSP70 protein, and EIII domain (residues 217–276 and 913–934) are shown by light gray bars. In these analyses, the predicted intrinsic disorder scores above 0.5 are considered to correspond to the disordered residues/regions.

The existence of four independent domains in the HSP70/EIII chimeric protein was further confirmed by the computational analysis of its intrinsic disorder predisposition. Figure 5 represents the results of this analysis and shows that HSP70/EIII has four predominantly ordered regions (residues 20–202, 332–530, 541–769, and 938–1080) that corresponded to the DsbC domain, two independent domains of HSP70, and the EIII domain. In the HSP70/EIII chimeric protein, the DsbC domain, HSP70 protein, and EIII domain are positioned at residues 1–216, 277–912, and 935–1080, respectively. Curiously, artificial linkers introduced to connect constituents of the HSP70/EIII chimeric protein (residues 217–276 and 913–934 between the DsbC domain and HSP70 and between HSP70 and EIII, respectively) were noticeably shorter than the predicted disordered segments in the corresponding regions (residues 203–331 and 770–937), indicating that both the N- and C-terminal tails of HSP70 are significantly disordered. 

Presumably, one can use a correlation between the sizes of the ordered regions and the transition enthalpy values to assign observed calorimetric peaks to actual domains of the protein. Based on this hypothesis, it is likely that the first transition with the Δ*H*1 of 99.5 kcal/mol corresponds to the melting of the ordered core of the N-terminal domain of HSP70 (residues 332–530), the second transition with the Δ*H*2 of 79.9 kcal/mol corresponds to the melting of the core of the EIII domain (residues 938–1080), and the third transition with the Δ*H*3 of 116 kcal/mol describes the melting of the ordered core of the C-terminal domain of HSP70 (residues 541–769).

As shown in Figure 4 and Table 2, all peak maximum temperatures (*T*_m_1–*T*_m_4) and therefore the thermal stability of all the HSP70/EIII domains increased in the presence of MGDG from *U. lactuca*, which forms a lipid matrix for antigen incorporation into the TI-complex [43]. Based on the remarkable changes in the *T*_m_2 and transition enthalpy Δ*H*2 values, the glycolipid environment had a particularly strong effect on the EIII conformation.

**Figure 6:**
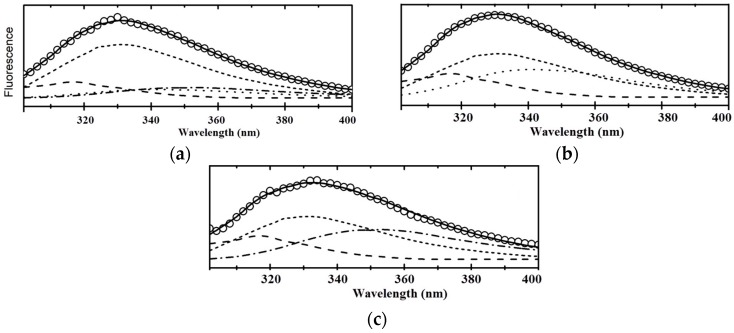
Fitting of the experimental fluorescent spectra data (symbols) of the HSP70 (**a**), HSP70/EIII (**b**) and, HSP70/EIII + MGDG. (**c**) to the theoretical model of the discrete states of tryptophan residues in proteins [45] (continues lines) which are sums of the following spectral components: S (dashed line); I (short dashed line); II (dotted line); III (dash-dotted line).

The deconvolution of the experimental fluorescence spectra of HSP70/EIII into elementary components corresponding to the emission of tryptophan fluorophore [44] (Figure 6) revealed the strong effect of *U. lactuca* MGDG on the HSP70/EIII tertiary structure. This conclusion follows from the noticeable changes in the contributions of different spectral forms to the total fluorescence (Table 3).

This analysis revealed that the main changes occurred in the contents of forms II and III. The spectrum of HSP70/EIII was characterized by the absence of form III, which disappeared as a result of the joining of EIII to HSP70. On the other hand, the addition of glycolipid not only restored this spectral form, but even increased the contribution of form III in the spectrum of HSP70/EIII at the expense of the total disappearance of form II. These rearrangements indicate the existence of a relaxing lipid effect on the protein tertiary structure, which contributes to the “correct” presentation of antigenic determinants and is probably the reason for the significant increase in the production of antibodies against EIII after mice immunization with the HSP70/EIII incorporated in the TI-complex in comparison with the effects of HSP70/EIII alone.

## 4. Conclusions

Earlier, we demonstrated the high adjuvant efficacy of the TI-complexes in relation to isolated and recombinant antigens [9,10,11,12]. The present work confirms the efficiency of the TI-complexes and testifies that using HSP70 in the content of the chimeric HSP70/EIII protein allows the effective presentation of the main antigenic domain of the TBEV envelope protein to the immune system, whereas the incorporation of HSP70/EIII in the TI-complex contributes to the further enhancement of the immune response to TBEV infection. The dependence of the HSP70 binding to lipid membranes on lipid composition [40] urges the study of the adjuvant effect of TI-complexes based on MGDG isolated from different marine macrophytes on HSP70/EIII immunogenicity. Based on the corresponding comparative studies, the most effective TI-complex(es) may be found for anti-TBE subunit vaccine construction.

## Figures and Tables

**Table 1:** Primers and their sequences.

Primer	Sequence
dnaK-X-Nco-dir	5′-ATATCCATGGCGATGGGTAAAATTATTGGTATCGAC-3′
dnaK-X-Sac-rev	5′-TATAGAGCTCGCTTTTTTGTCTTTTACTTCTTCGAATTC-3′
E3-X-Sac-L-dir	5′-TATAGAGCTCGGGTGGTGGTGGTTCTGGTGGTGGTGGTTCTGGTGGTGGTGGTTCTGGTCTTACATACACAATGTGCGACAAGACGAAATTCAC-3′
E3-X-Sal-stop-rev	5′-TATA GTCGAC TTA GTTAAAGGCACCGCCAAGAACTGT-3′

**Table 2:** Arrhenius equation parameter values for peaks obtained in result of deconvolution of differential scanning calorimetry (DSC) thermograms of HSP70, HSP70/EIII alone and HSP70/EIII + MGDG from *Ulva lactuca*.

	Hsp70	HSP70/EIII	HSP70/EIII + MGDG *U. lactuca*
*T*_m_1, °C	54.6 ± 0.1	54.2 ± 0.1	55.4 ± 0.1
Δ*H*1, kcal/mol	92.1 ± 0.7	99.5 ± 1.7	96.9 ± 1.4
*T*_m_2, °C	-	58.4 ± 0.3	63.2 ± 0.1
Δ*H*2, kcal/mol mol^−1^	-	79.9 ± 1.5	103.0 ± 1.9
*T*_m_3, °C	66.4 ± 0.2	67.1 ± 0.1	67.7 ± 0.1
Δ*H*3, kcal/mol	81.5 ± 1.5	116.0 ± 1.5	170.0 ± 1.9
*T*_m_4, °C	68.5 ± 0.4	70.1 ± 0.6	71.3 ± 0.5
Δ*H*4, kcal/mol	129.0 ± 1.3	124.0 ± 1.9	126.0 ± 2.6

*T*_m_—peak maximum temperature, Δ*H*—enthalpy of transition. The data are expressed as the mean ± average deviation of three separate determinations.

**Table 3:** Contribution to the fluorescence emission of the spectral forms (%), according to the model of the discrete states of tryptophan residues in proteins

Protein	Spectral Forms (%)			
	S	I	II	III
HSP70	13.6 ± 0.3	58.5 ± 0.5	12.2 ± 0.3	15.7 ± 0.2
HSP70/EIII	19.5 ± 0.5	45.7 ± 0.4	34.8 ± 0.5	0
HSP70/EIII + MGDG *U. lactuca*	19.3 ± 0.5	42.8 ± 0.5	0	37.9 ± 0.4

The data are expressed as the mean ± average deviation of three separate determinations.
